# A Novel Subspace Alignment-Based Interference Suppression Method for the Transfer Caused by Different Sample Carriers in Electronic Nose

**DOI:** 10.3390/s19224846

**Published:** 2019-11-07

**Authors:** Zhifang Liang, Fengchun Tian, Ci Zhang, Liu Yang

**Affiliations:** 1 School of Communication and Information Engineering, Chongqing University of Posts and Telecommunications, Chongwen Road 2nd, Nan’an District, Chongqing 400065, China; yangliu@cqupt.edu.cn; 2 School of Microelectronics and Communication Engineering, Chongqing University, 174 ShaZheng Street, ShaPingBa District, Chongqing 400044, China; fengchuntian@cqu.edu.cn (F.T.); ZhangCi@cqu.edu.cn (C.Z.)

**Keywords:** electronic nose, subspace alignment, interference suppression, transfer

## Abstract

A medical electronic nose (e-nose) with 31 gas sensors is used for wound infection detection by analyzing the bacterial metabolites. In practical applications, the prediction accuracy drops dramatically when the prediction model established by laboratory data is directly used in human clinical samples. This is a key issue for medical e-nose which should be more worthy of attention. The host (carrier) of bacteria can be the culture solution, the animal wound, or the human wound. As well, the bacterial culture solution or animals (such as: mice, rabbits, etc.) obtained easily are usually used as experimental subjects to collect sufficient sensor array data to establish the robust predictive model, but it brings another serious interference problem at the same time. Different carriers have different background interferences, therefore the distribution of data collected under different carriers is different, which will make a certain impact on the recognition accuracy in the detection of human wound infection. This type of interference problem is called “transfer caused by different sample carriers”. In this paper, a novel subspace alignment-based interference suppression (SAIS) method with domain correction capability is proposed to solve this interference problem. The subspace is the part of space whose dimension is smaller than the whole space, and it has some specific properties. In this method, first the subspaces of different data domains are gotten, and then one subspace is aligned to another subspace, thereby the problem of different distributions between two domains is solved. From experimental results, it can be found that the recognition accuracy of the infected rat samples increases from 29.18% (there is no interference suppression) to 82.55% (interference suppress by SAIS).

## 1. Introduction

**Figure 1:**
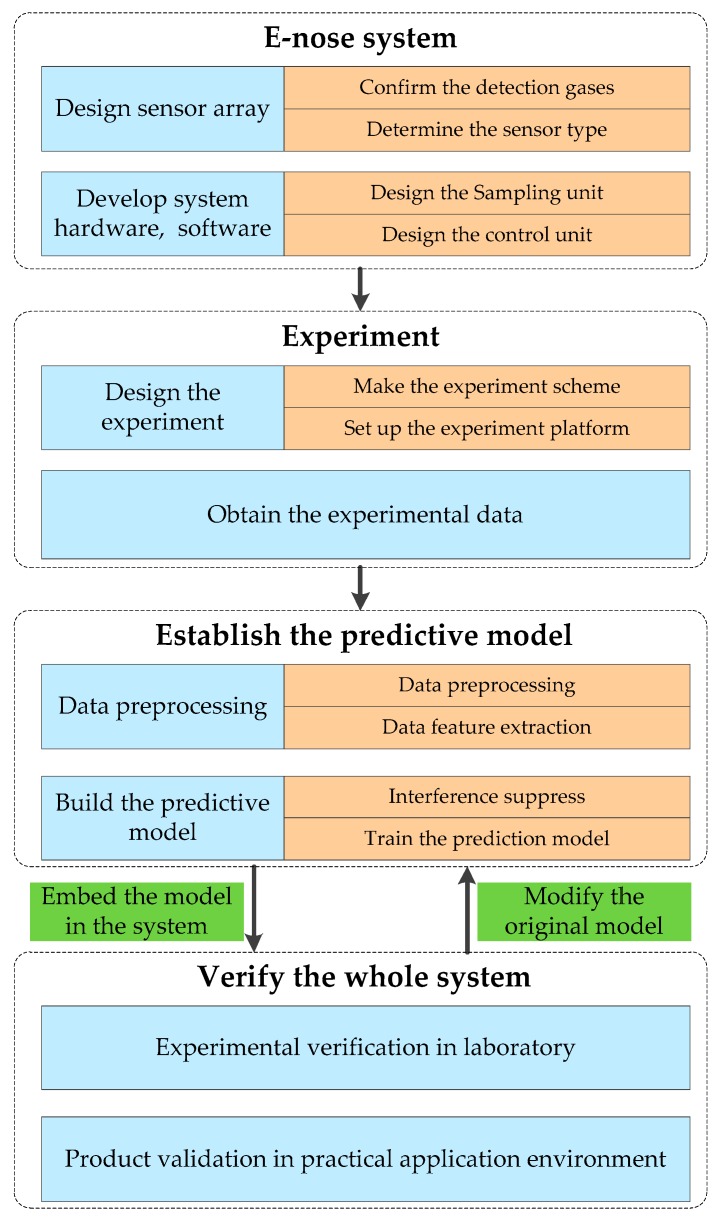
The design flow diagram of the e-nose system.

As an artificial nose, electronic nose (e-nose) can analyze the gas characteristics quickly by simulating the biological olfactory system. It consists of a gas sensor array and an appropriate pattern recognition system. The basic structure of an e-nose system is similar to that of the biological olfactory system, in which the gas sensor array, the signal processing unit and the pattern recognition unit respectively simulate the olfactory receptor, the olfactory bulb and the cerebral cortex in the biological olfactory system of the mammal [1]. Due to its portability and ease of use, e-nose technology is superior to other detection methods, such as chemical detection methods, gas chromatography and mass spectrometer, etc. [2]. With the development of sensing technology and intelligent algorithms, e-nose technology has been widely used in many areas, such as disease diagnosis by detecting specific biomarkers of diseases or bacteria [3,4,5], air quality monitoring by analyzing the gas pollutions [6,7,8], tea quality assessment [9,10], food quality detection [11,12], etc. For a specific application, the design flow of the e-nose system is shown in Figure 1:

As the collector of odor information, the choice of sensor is crucial, so the selected sensors should have good cross-sensitivity, selectivity, reliability and robustness. Objectively, there are some intrinsic flaws during the production and use of the sensors. These flaws make the e-noses face some interference problems, such as background interference, transfer among multiple instruments, sensor drift, etc. The performance of e-nose system will deteriorate when it suffers from interferences. Some corresponding solutions have been proposed to eliminate or suppress these interferences. The proposed methods can be divided into the following four categories: (1) Methods based on the traditional correction models, which suppress the interference by directly correcting the responses of the sensors, such as: single variable correction [13], multivariate correction [14], mapping [15], regression [16], orthogonal signal correction algorithm (OSC) [17,18], etc.; (2) methods based on the component analysis algorithm, such as: principal component analysis (PCA) [19], independent component analysis (ICA) [20], etc.; (3) methods based on adaptive estimation model, such as adaptive self-organizing map [21], multiple self-organizing maps [22], etc.; (4) methods based on the idea of transfer learning, such as windowed piecewise direct standardization (WPDS) [23], domain adaptation extreme learning machine (DAELM) [24], drift correction autoencoder (DCAE) [25], etc. These suppression methods have achieved a certain effect on the above three types of interferences.

However, during the research of e-nose technology, especially for the development of medical e-nose system for the wound infection detection, another key problem is more worthy of attention, i.e., the prediction accuracy drops dramatically when the prediction model established by laboratory data is directly used in human clinical samples. For a type of bacteria, the host (i.e., carrier) can be the culture solution, the animal wound, or the human wound. Bacterial culture solution or animals (such as: mice, rabbits, etc.) are usually used as experimental subjects to collect sufficient sensor array data to establish the robust predictive model. However, the detection effect will be poor when the prediction model established by the bacterial culture solution (or animal) experimental data is directly used for human detection, which may be caused by the statistical distribution difference of the sensor array data collected under different kind of sample carriers. So in the practical application, another serious interference problem has appeared, i.e., background interference caused by the different carriers, which make the data distribution different and the prediction performance of the e-nose poor. Different sample carrier, different background interference, and it can be called “transfer caused by different sample carriers”. This transfer caused by different sample carriers can be considered as a kind of interference which makes the research of medical e-nose into a bottleneck. Further research on the suppression of this interference is necessary.

For the transfer caused by different sample carriers, there are few feasible methods currently, but the suppression method can refer to the methods for background interference suppression. In Ref. [17,26], mice were used as experimental subjects in the e-nose system for wound infection detection, and the background (i.e., the smells of mice bodies) was very strong. Wavelet transform, ICA and OSC algorithm were used to suppress the background interference. In these methods, an extra reference vector was necessary to determine the interference component, which was time consuming and difficult in the complex real-word applications. Therefore, these methods are not ideal ways to suppress the background interference, and they are not the good solutions for the transfer caused by different sample carriers.

For the transfer caused by different sample carriers, traditional machine learning methods are not feasible due to their weak generalization to the data with different distributions. The interference makes the distribution of sensor array responses different between the two kinds of sample carriers, so it is a challenging problem in machine olfaction community and sensory field. However, the transfer learning [27], which tries to transfer the knowledge from some previous tasks to a target task and can solve the problem of distribution difference, is an appropriate method for suppressing the transfer caused by different sample carriers.

The subspace is a space spanned by the eigenvectors that contains the domain invariant features of the data domain. In Ref. [28], manifold alignment aligned two datasets from two different manifolds such that they can be projected to a common subspace. In Ref. [29], some subspaces were generated along the geodesic path connecting the source subspace and the target subspace on the Grassmann manifold. Then, projected source and target onto points along that path. In Ref. [30], Gong et al. proposed a geodesic flow kernel which modeled incremental changes between the source and target subspaces. In these two papers, a set of intermediate subspaces were used to model the shift between the two domains. In Ref. [31], subspace alignment was proposed, which aligned the subspaces by computing the linear map that minimized the Frobenius norm of the difference between them. In Ref. [32], the work sought a domain invariant feature space by learning a mapping function which aligned the source subspace with the target one. In these two papers, the proposed methods do not construct a set of intermediate subspaces. Subspace Distribution Alignment (SDA) approach was proposed to improve domain alignment by taking the difference of the source and target distributions into account [33]. A new framework for transfer where the source and target domains were represented by subspaces described by eigenvector matrices was introduced [34]. In recent years, subspace alignment with domain correction capability and transfer learning idea has been extensively studied. However, it is rarely used for interference suppression in e-nose. Based on the idea of subspace alignment and the characteristics of the interference, subspace alignment is suitable for the study of interference suppression in e-nose system, since compared to the training data, the distribution of the collected data changes when the e-nose system is facing interference.

Motivated by the fact that subspace alignment can be used to transfer the knowledge between two domains and the distribution of sensor data will be changed with different carrier, a novel subspace alignment-based interference suppression (SAIS) method with domain correction capability is proposed to solve the transfer caused by different sample carriers. This method consists of two steps: (1) Get the subspace for each domain; (2) move these two subspaces closer to reduce the discrepancy between two domains. In this paper, a designed e-nose system is used for detection of bacteria in wound infection, and the sensor data obtained by the culture solution as carrier is set as source domain while the sensor data obtained by the rats as carrier is set as target domain.

The rest of this paper is organized as follows. The experimental setup, experiment procedure and experimental data are introduced in Section 2. The proposed SAIS method is introduced in Section 3. Experimental results and discussion are presented in Section 4. Conclusion is given in Section 5.

## 2. Experiments

The designed e-nose system is used for detection of bacteria in wound infection. There are many kinds of bacteria in the wound, and Acinetobacter baumannii, Escherichia coli, Staphylococcus aureus, Pseudomonas aeruginosa and Enterobacter cloacae are the most common bacteria in wound infection [35,36,37]. The volatile organic compounds (VOCs) produced by these bacteria are different in the process of metabolism [35]. So, it is feasible to detect the bacteria using e-nose system by identifying these VOCs, which was confirmed in some literature [38,39,40]. Next, the designed e-nose system, the experiment and materials, and experimental data will be introduced in detail as follows.

### 2.1. Experimental Setup

**Figure 2:**
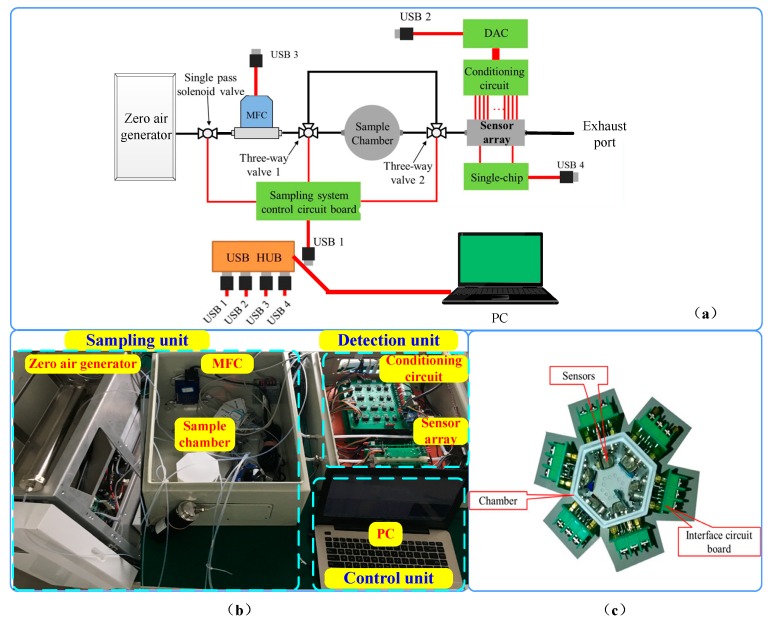
Schematic diagram of e-nose designed for detection of bacteria (**a**), photo of the experimental platform (**b**) and photo of the sensor chamber (**c**).

The schematic diagram of e-nose system is introduced in Figure 2a, and the picture of real products is shown in Figure 2b. The e-nose system consists of three parts: a sampling unit, a detection unit and a control unit. The sampling unit consists of 2 three-way valves, a sample chamber, and a mass flow controller (MFC: CS200-C, Beijing Sevenstar Electronics Company Ltd., Beijing, China). The sensor array and its corresponding conditioning circuit constitute the detection unit. The control unit is composed of a PC equipped with a specific software system, which is used to control the sampling system, collect data and analysis the sample. The experimental platform is improved on the basis of the experimental platform in Ref. [26], and the following adjustments have been made: (1) The clean air is replaced by zero air produced by the specific generator as carrier gas. Since the useful signal is weak in the animal experiment, the pure carrier gas is needed. (2) The airflow of the experimental platform changes from negative pressure to positive pressure and the air pump is removed. The clean air is used as the carrier gas in Ref. [26], and at the end of the system, the pump is added to achieve the gas circulation of the whole platform without affecting the purity of the carrier gas due to the air pump. In this paper, the zero air produced by the specific generator is used as carrier gas. The gas generator has a certain gas pressure when it generates gas, which can make the gas flow, so the pump is not needed. (3) The position of the MFC has changed. In order to control the flow rate of the gas entering the sample chamber, the MFC is placed between the gas generator and the sample chamber in this paper. If the MFC is still placed behind the sensor chamber as in Ref. [26], due to the high pressure of the gas generated by the gas generator, the high air tightness of the sample chamber and the sensor chamber is needed.

The photo of the sensor chamber is shown in Figure 2c. There are 31 gas sensors and a module with three sensors for temperature, humidity and atmospheric pressure in the chamber. The main types of metabolites of the five tested bacteria are hydrocarbons, ketones, alcohols, fatty acids, aldehyde, esters, phenols, benzene ring compounds, sulfur compounds, nitrogenous compounds, etc. The gas sensors are selected according to their sensitivities to these metabolites, and the detailed information of these gas sensors is shown in Table 1. In order to minimize the volume of the sensor chamber with all sensors installed, the sensor chamber is designed as a hexagonal prism and the sensors are fixed on the surface of the prism. To reduce the adsorption of the gas chamber, the material of chamber is Teflon.

### 2.2. Materials and Experiment

#### 2.2.1. Materials

**Ethics Statement**. All animal care and use protocols in this study were performed in accordance with the Regulations for the Administration of Affairs Concerning Experimental Animals approved by the State Council of the People’s Republic of China. All animal experiments in this study were approved by the Animal Ethical and Experimental Committee of Chongqing University in accordance with their rules and regulars. All surgery was performed under sodium pentobarbital anesthesia, and all efforts were made to minimize suffering.

**Materials**. In this paper, five kinds of bacteria, i.e., Acinetobacter baumannii, Escherichia coli, Staphylococcus aureus, Pseudomonas aeruginosa and Enterobacter cloacae, which are the most common bacteria in the wound, are detected by the e-nose. The carrier of the bacteria can be the culture medium, the animal wound. Different sample carriers, different background interference. The experimental data in this paper are collected by making the culture medium and animal wounds as carriers.

In the experiment, the culture mediums are broth medium. As well, the Sprague–Dawley (SD) male rats, 6–8 weeks old and 225–250 g weight, are used as experimental subjects. During the experiment, a small incision (about 3 cm long) is made in the back of rat near its hips after it is anaesthetized. Then 1 mL bacterial solution (10^9^ CFU/mL) is injected into the wounds of rats. During the experiment, the same volume and concentration of the bacterial solution are injected into the wound of rat each time to ensure the consistency of bacterial concentration as much as possible. Use a cotton swabs to collect pus from the infected rat wounds, and the cotton swabs is as a sample. Starting from 24 h after infection in rats, the infected pus samples are collected every 24 h, and collected a total of five times.

#### 2.2.2. Experiment

**Figure 3:**
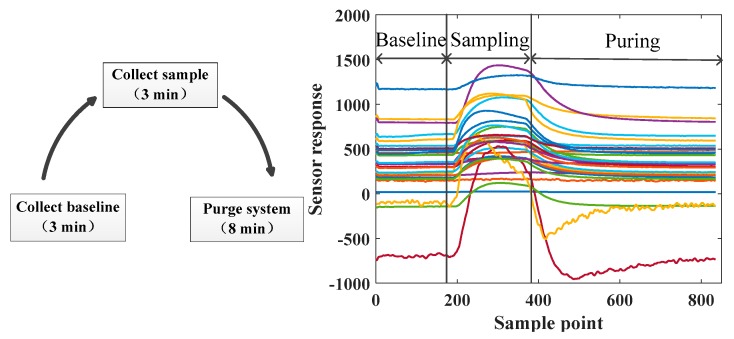
Illustration of sampling process and corresponding sensor response cycle.

In experiments, gas flow of MFC is set up to 100 mL/min. The sampling process of designed e-nose system is divided into three steps: baseline collecting, sample collecting and system purging. The total time of each experiment is 14 min including 3 min for baseline collecting, 3 min for sample collecting and 8 min for system purging. The sampling process and corresponding sensor response cycle are shown in Figure 3. The sampling frequency is set to 1 Hz. For each sample, 840 sampling points are collected. The carrier gas is zero air produced by the specific generator.

The experiment procedure is the same as in Ref. [26].

(1) Start the experimental setup.
(2) Preheat 45 min, and meanwhile let the sensors be recovered by thoroughly exposing the sensor array to clean air.
(3) Put the sample in culture dish, and rest for 10 min.
(4) Put the culture dish in sample chamber and close the sample chamber.
(5) Collect baseline.
(6) Collect samples.
(7) Purge the e-nose system using cleaning air.
(8) Open the sample chamber, and remove the culture dish.
(9) Test the next sample, and repeat steps (3)–(8).

### 2.3. Experimental Data

The datasets are prepared respectively, as follows.

In this paper, the corresponding relationship between the sample sequence number and the bacteria is as follows: Bacteria 1: Acinetobacter baumannii, Bacteria 2: Escherichia coli, Bacteria 3: Staphylococcus aureus, Bacteria 4: Pseudomonas aeruginosa, Bacteria 5: Enterobacter cloaca.

Dataset 1: Dataset 1 shown in Table 2 is a multi-class sample set composed of five kinds of bacteria samples, and the carrier of each bacterium is the culture medium, i.e., the bacterial culture solution is directly used as a sample for experiment. Each sample is the maximum value in the sensor responses sequence. The experimental process is illustrated in Section 2.2.2. The sensor response can reflect the characteristics of each type of bacteria more accurately with the culture solution as the carrier since its background interference is less, thus this dataset can be used to train a robust classification model. The dataset is often divided into training data and testing data. Kennard–Stone sequential (KSS) method [41,42] is used to select the training samples from Dataset 1. In general, the proportion of training and testing data is approximately 2:1.

Dataset 2: Dataset 2 shown in Table 2 is a multi-class sample set composed of five kinds of bacteria samples, and the carrier of each bacterium is the infected rats. The sample collection process is introduced in Section 2.2.1. Animal experiments are carried out four times. In the first, second, and fourth experiments, ten rats are randomly divided into five groups (two in each group) and infected with Acinetobacter baumannii, Escherichia coli, Staphylococcus aureus, Pseudomonas aeruginosa, and Enterobacter cloaca, respectively. In the third experiment, fifteen rats are randomly divided into five groups (three in each group) and infected with five bacteria respectively. Use a cotton swabs to collect pus from the infected rat wounds. Starting from 24 h after infection in rats, the infected pus samples are collected every 24 h, and collected a total of five times. The number of samples obtained for each type of bacterium is shown in Table 2. Each sample is the maximum value in the sensor responses sequence. The experimental process is illustrated in Section 2.2.2.

## 3. Proposed Method

Suppose that the source domain and target domain are represented by **X**_S_ and **X**_T_, respectively, where $X_{S}=[x_{S}^{1},\cdots ,x_{S}^{N_{S}}]\in {ℜ}^{d\times N_{S}}$ and $X_{T}=[x_{T}^{1},\cdots ,x_{T}^{N_{T}}]\in {ℜ}^{d\times N_{T}}$. In this paper, we assume that all the samples in the source domain are labeled data while all the samples in the target domain are unlabeled data. The corresponding labels of source data are $T_{S}=[t_{S}^{1},\cdots ,t_{S}^{N_{S}}]$. As well, *d* is the dimension of the sample feature, *N*_S_ and *N*_T_ is the number of samples in source domain and target domain. The distributions of these two data sets are different but related (for example, datasets collected by the same sample under different carriers, datasets collected by the same sensor array for the same gases at different time). In order to make the prediction model trained by the source data can be used for target data, the distribution of target data must be similar to the source domain data. Therefore, it is necessary to make some transformation to the target data. 

### 3.1. Subspace Alignment-Based Interference Suppression Method (SAIS)

**Figure 4:**
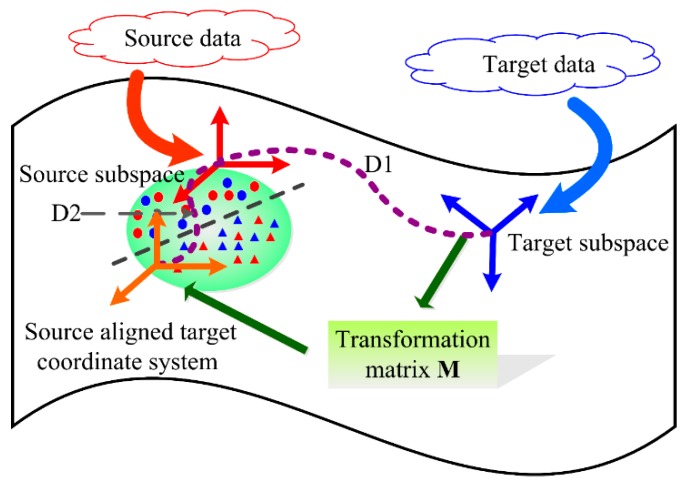
Schematic diagram of the target subspace aligned to the source subspace (Note: Source domain is represented by the source subspace **S**_S_ and target domain is represented by the target subspace **S**_T_. Then the target subspace is aligned to the source subspace by **M**, which makes the target subspace close to the source space in the Bregman divergence perspective (i.e., D2 < D1). Then the source data is projected to the source subspace and the target data is projected to the source-aligned target subspace. As well, the prediction model trained by projected source data can be used to the projected target data.).

The idea of subspace alignment is used to solve the problem of different distribution between source data and target data in this paper. Schematic diagram of the target subspace aligned to the source subspace is shown in Figure 4.

The source and target domains are represented by subspaces spanned by eigenvectors. Suppose that the subspace of source domain **X**_S_ and target domain **X**_T_ are represented by **S**_S_ and **S**_T_, respectively, and **S**_S_$\in {ℜ}^{d\times D}$, **S**_T_$\in {ℜ}^{d\times D}$, *D* is the size of subspace. These two subspaces satisfy the following conditions:

(1) $${\left(S_{S}\right)}^{T}S_{S}=I_{D}\;\mathrm{and}\;{\left(S_{T}\right)}^{T}S_{T}=I_{D}$$

where **I***_D_* is the identity matrix of size *D*, (∙)^T^ denotes the transpose operator. **M** is learned by minimizing the following Bregman matrix divergence *F*(**M**) (i.e., minimize the difference between source domain and target domain) [31]:

(2) $$F(M)=||S_{S}-S_{T}M|{|}_{F}^{2}$$

(3) $$M*=\arg\min_{M}(F(M))$$

where $||\cdot |{|}_{F}^{2}$ is the Frobenius norm. It is not necessary to add a regularization term in the Equation (2), since **S**_S_ and **S**_T_ are generated from the first *D* eigenvectors which are intrinsically regularized. So it is thus possible to obtain a simple solution of Equation (3) in closed form. Due to the Frobenius norm is invariant to orthonormal operations [31], Equation (2) can be written as follows:

(4) $$F(M)=||S_{S}-S_{T}M|{|}_{F}^{2}=||{\left(S_{T}^{}\right)}^{T}S_{S}-{\left(S_{T}^{}\right)}^{T}S_{T}M|{|}_{F}^{2}=||{\left(S_{T}^{}\right)}^{T}S_{S}-M|{|}_{F}^{2}$$

From Equation (4), we can conclude that the optimal M* can be obtained as [31]:

(5) $$M*={\left(S_{T}^{}\right)}^{T}S_{S}$$

For the target domain, the new coordinate system $S_{a}$ is transformed to $S_{a}=S_{T}M^{*}=S_{T}{\left(S_{T}\right)}^{T}S_{S}$. As well, $S_{a}$ can be called as the source-aligned target coordinate system. If the source domain and target domain are the same, then **S**_S_ = **S**_T_ and M* is the identity matrix.

It is crucial to accurately obtain the subspace for each domain in this method. In this paper, principle component analysis (PCA) is used to obtain the subspace. First, every sample of source data and target data is transformed to a *d*-dimensional *z*-normalized vector (i.e., zero mean and unit standard deviation). It is an important step in most of the subspace-based domain adaption methods [43,44]. Then, the source data and target data are analyzed by PCA respectively, and the *D* eigenvectors corresponding to the *D* largest eigenvalues are used as the bases of the source and target subspaces. *D* is the size of subspace. The obtained subspaces satisfy the conditions in Equation (1). 

**Figure 5:**
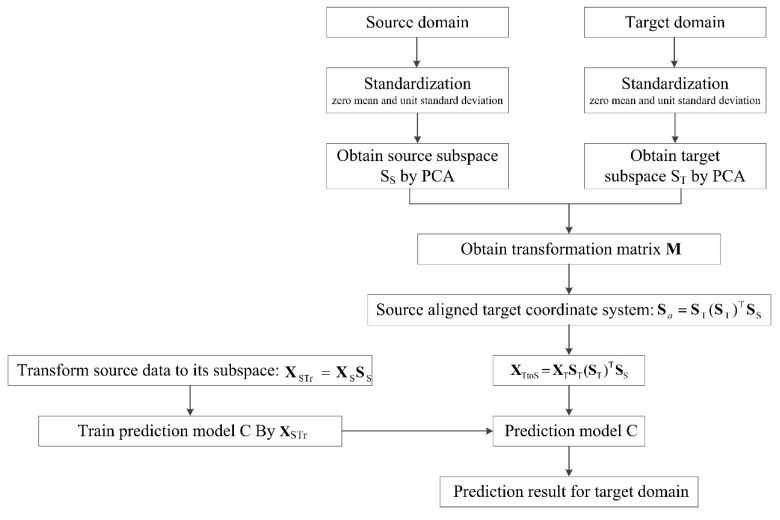
Flow chart of SAIS method.

Subspace alignment can be used to solve the problem of different distribution of two domains, so it can be regarded as a kind of domain correction method. Motivated by the different distribution of data collected under different bacterial carriers and the idea of subspace alignment, a novel subspace alignment-based interference suppression (SAIS) method for suppressing the transfer caused by different sample carriers in e-nose is proposed. The procedure of SAIS algorithm is introduced in Table 3, and the flow chart is illustrated in Figure 5.

### 3.2. Merits of the Proposed SAIS Method

The merits of the proposed SAIS method are as follows:

(1) Most of interference suppression methods are from the perspective of component correction (such as PCA, ICA, OSC, etc.). The proposed SAIS method is from the perspective of transfer learning, especially from the perspective of subspace alignment, to realize the knowledge transfer for interference suppression;

(2) The proposed SAIS method directly reduces the difference between two domains by moving the source and target subspace closer. As well, it projects the source data to the source subspace and the target data to the target subspace instead of methods that project source data to the target subspace or target data to the source subspace;

(3) The method only requires source label information and does not require any target label information, so it is an unsupervised method; 

(4) By aligning the source and target subspaces, the proposed method is intrinsically regularized, i.e., there is no need to add a regularization term and tune the corresponding parameters in the objective;

(5) The proposed SAIS method aligns the two subspaces directly, instead of computing a large number of intermediate subspaces which may potentially be a costly tuning procedure and time consuming.

## 4. Results and Discussion

### 4.1. Data Preprocessing

In this paper, the Butterworth low-pass filter with the cut-off frequency of 0.1 Hz is used to make the response curve smooth and reduce some noises. The *z*-score method is used to standardize the source dataset and target dataset. The formula for *z*-score method is as follow:

(6) $$X(i)=\frac{x_{i}-mean(x)}{std(x)},i=1,2,\cdots ,n$$

where $x={\left[x_{1},x_{2},\cdots ,x_{n}\right]}^{T}$ is a feature sequence for one sensor in a dataset, and the length is *n*. mean(⋅) and std(⋅) are the functions for calculating the mean and standard deviation of a sequence, respectively. $X={\left[X(i),\cdots ,X(n-20+1)\right]}^{T}$ is the normalized signal sequence for one sensor. The *z*-score method is based on the mean and standard deviation of the original data and the normalized data conforms to the standard normal distribution, i.e., the mean is 0 and the standard deviation is 1.

### 4.2. Comparison of Data Distribution 

**Figure 6:**
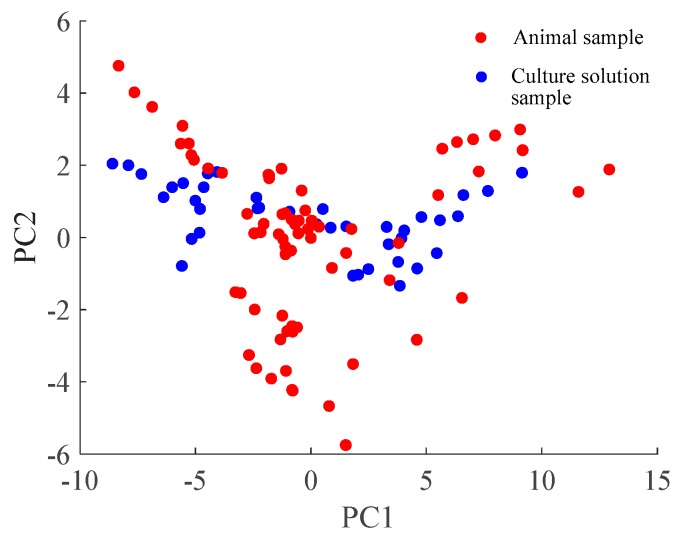
Space distribution of two dataset with different sample carrier.

To observe the space distribution difference between two datasets with different sample carrier, the PCA algorithm is applied to Dataset 1 and Dataset 2. The first principal component (PC1) is the horizontal axis and the second principal component (PC2) is the vertical axis. The projected 2-D subspace for the same kind of bacteria with different carrier is shown in Figure 6, from which the significant changes of data space distribution can be observed.

In this paper, the sample obtained by using the culture solution as the carrier (i.e., the Dataset 1) is set as source domain while the sample obtained by using the infection rats as the carrier (i.e., the Dataset 2) is set as target domain. The sensor response of Dataset 1 can reflect the characteristics of each type of bacterium since its background interference is less, thus this dataset can be used to train a robust classification model. As well, the target subspace is aligned to the source subspace by the proposed SAIS method which makes the trained prediction model can be used for the prediction of the target samples.

### 4.3. Discriminant Results and Comparisons

In order to verify the effectiveness of proposed SAIS method, the discriminant performance of the target data is used as the evaluation criterion, which is predicted by the prediction model trained by source data. As well, the Extreme Learning Machine (ELM) is used as the discrimination method, which is a common and time-saving method.

For transfer caused by different sample carriers, there are few feasible methods currently, therefore some common interference suppression methods are used as comparison methods to verify the effectiveness of proposed SAIS method. The comparison methods are as follows:

(1) There is no interference suppression, i.e., prediction model trained by the source data is directly used for target data prediction. Support Vector Machine (SVM) and ELM are select as the classification method, due to these two methods are the commonly used classification methods;

(2) The principle component analysis algorithm (PCA) is a classical method for interference suppression in e-nose. So PCA can be directly used to suppress the transfer caused by different sample carriers. Also, SVM and ELM are used as the classification method;

(3) The orthogonal signal correction (OSC) algorithm is a classical method for interference suppression in e-nose. So it can be used to suppress the transfer caused by different sample carriers.

(4) Domain regularized component analysis (DRCA) proposed by L. Zhang, et al. [45] is used to suppress the sensor drift, and it is based on the idea of transfer learning. In this paper, it is used to suppress the transfer caused by different sample carriers in e-nose;

(5) Domain correction and adaptive extreme learning machines (DC-AELM) method proposed by Z. Liang, et al. [46] is used to suppress the sensor drift and background interference, and it realize the knowledge transfer for interference suppression from the perspective of domain correction and decision-making. In this paper it is used as a comparison method;

(6) Domain Adaptation Extreme Learning Machines (DAELM) proposed by L. Zhang, et al. [24] is used to compensate the sensor drift. Source DAELM (DAELM-S) and Target DAELM (DAELM-T) were proposed to suppress the drift by leveraging a limited number of labeled data from target domain. DAELM-S learns a robust classifier based on the source domain by leveraging a limited number of labeled samples from target domain. DAELM-T learns a classifier based on a limited number of labeled data in target domain by leveraging a prelearned base classifier in source domain.

**Figure 7:**
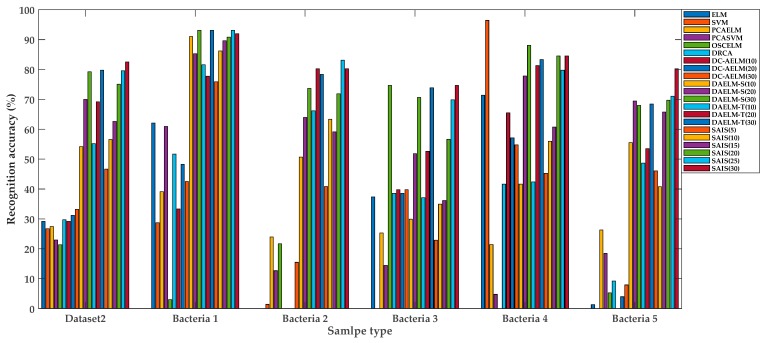
Comparisons of different methods for each bacterium.

The recognition accuracies of every method are shown in Table 4. The first column is the method for interference suppression. The second column is the recognition accuracy of the test set from Dataset1 (Dataset 1 is used to train the prediction model). The third column is the overall recognition rate of Dataset 2, and the rest is the recognition rate for each kind of bacterium from infected rats in Dataset 2. For DC-AELM method, the recognition accuracies of different number of transfer samples are given (i.e., DC-AELM(10) indicates that there are 10 transfer samples for DC-AELM method, and the rest is similar to it). For DAELM-S and DAELM-T methods, the recognition accuracies of different number of labeled target samples are given (i.e., DAELM-S(10) indicates that there are 10 labeled target samples for DAELM-S method, and the rest is similar to it). As well, for the proposed SAIS method, the recognition accuracies for different number of target samples used to calculate the target subspace are given (i.e., SAIS(5) indicates that there are 5 target samples for SAIS method, and the rest is similar to it). The size of the subspace in SAIS method is 25. The performances of all methods are illustrated in Figure 7. The observations are as follows.

**Figure 8:**
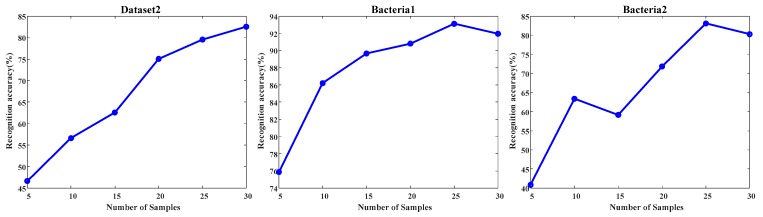

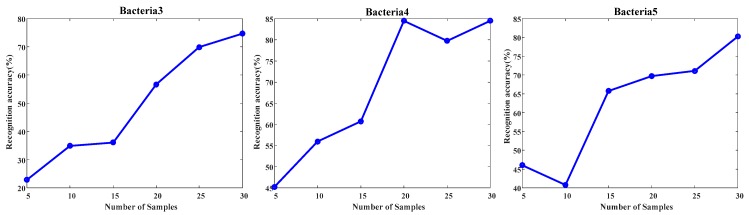
Recognition accuracy (%) with respect to different number of target samples used to calculate the target subspace.

(1) It can be seen from Table 4 that, in the absence of interference suppression, the recognition rate of target domain is very poor which cannot meet the actual application requirements. This is due to the difference in the distribution of source data and target data and the poor generalization of traditional machine learning methods.

(2) From Table 4 and Figure 7, it can be found that the proposed SAIS method is superior to other methods in discrimination performance. As well, the discrimination performance for each bacterium is very good. However, there is a special case. For bacteria 4, the recognition rate of SVM without suppressing interference is highest. However, the recognition rate of SVM for other bacteria, especially the overall recognition rate of Dataset 2 is very low. Therefore, it is not feasible to directly discriminate using SVM.

(3) From Table 4 and Figure 7, it can be found that the proposed SAIS is slightly better than the DAELM (DAELM-S and DAELM-T), but it is not obvious. The proposed SAIS method does not use the label information of the target domain, while the DAELM uses that. This is one of the advantages of the proposed SAIS method.

(4) As shown in Figure 8, the accuracies of the proposed SAIS show an upward trend with the increase of target samples used to calculate the target subspace. Therefore, the number of target samples used to calculate the target subspace will be critical to the effect of interference suppression.

(5) From Table 4, the DCRA method and the DC-AELM method have little effect on transfer caused by different carrier. This may be because these methods only adjust the marginal distribution without adjusting the conditional distribution.

### 4.4. Discussions

The reasons behind the obvious improvements are as follows:

(1) From the perspective of subspace alignment, the difference between the two domains can be reduced by directly moving the source subspace and the target subspace closer. This is different from many other domain correction methods;

(2) The proposed method projects the source data to the source subspace and the target data to the target subspace instead of methods that project source data to the target subspace or target data to the source subspace;

(3) The SAIS method is intrinsically regularized, and it is not necessary to add a regularization term and tune the corresponding parameters, so there is no influence of the regularization parameter adjustment;

In summary, the SAIS method is used to align the target subspace to the source subspace, so that the source domain data transformed to the source subspace and the target domain data transformed to the source-aligned target coordinate system have the same distribution, thereby both can share the same predictive model.

## 5. Conclusions

In this paper, a novel subspace alignment-based interference suppression (SAIS) method is proposed to suppress the transfer caused by the different carrier in e-nose. The method is motivated from the different distribution of data collected under different bacterial carriers and the idea of subspace alignment. The merits of the proposed SAIS method are as follows: (1) From the perspective of the domain subspace, the distributions of target domain and source domain are corrected by aligning the subspaces of two domains. (2) The proposed method aligns the two subspaces directly, instead of computing a large number of intermediate subspaces. (3) The proposed method is intrinsically regularized, so there is no need to add a regularization term and tune the corresponding parameters. It can be seen from the experimental results that the proposed method has obvious effects on suppressing the transfer caused by different carrier. The recognition accuracy of the infected rat samples increases from 29.18% (there is no interference suppression) to 82.55% (interference suppress by SAIS). As well, the proposed method can also be used to solve other interference problems, such as: background interference, transfer among multiple system and sensor drift.

Next, the method for getting the subspace for each domain and the size *D* of the subspace of SAIS should be further studied by leveraging some machine learning method. As well, more effective interference suppression method may also be an interesting research direction in e-nose.

## Figures and Tables

**Table 1:** Main information of gas sensors in sensor array.

Sensor	Sensitive Substances	Type	Producer (Country)
TGS813	Hydrocarbons (methane, propane, isobutane, etc.), Alcohols (ethanol, etc.), Inorganic gases (hydrogen, carbon monoxide, etc.)	MOS	Figaro (Japan)
TGS816	Hydrocarbons (Methane, Propane, Isobutylene, etc.), Alcohols (Ethanol, Butanol, etc.), Inorganic gases (Hydrogen, Carbon monoxide, etc.)	MOS	Figaro (Japan)
TGS822	VOCs, Inorganic gases (Carbon monoxide, Hydrogen, etc.)	MOS	Figaro (Japan)
TGS826	Nitrogenous compounds (Ammonia, Amines, etc.), Alcohols (Ethanol, etc.), Hydrocarbons (Methane, Butane, etc.)	MOS	Figaro (Japan)
TGS2600	VOCs, Inorganic gases (Carbon monoxide, Hydrogen, etc.), Cigarette smoke	MOS	Figaro (Japan)
TGS2602	VOCs, Inorganic gases (Hydrogen sulfide, Ammonia, etc.)	MOS	Figaro (Japan)
TGS2610C	Alcohols (Ethanol, etc.), Hydrocarbons (Methane, Ethane, Propane, etc.), Inorganic gases (Carbon monoxide, Hydrogen, etc.)	MOS	Figaro (Japan)
TGS2610D	Similar to TGS2610C, adds an internal alcohol filter	MOS	Figaro (Japan)
TGS2611C	Alcohols (Ethanol, etc.), Hydrocarbons (Methane, Ethane, Propane, Butane, etc.), Inorganic gases (Carbon monoxide, Hydrogen, etc.)	MOS	Figaro (Japan)
TGS2611D	Similar to TGS2611C, adds an internal alcohol filter	MOS	Figaro (Japan)
TGS2620	VOCs, Inorganic gases (Ammonia, Hydrogen, etc.)	MOS	Figaro (Japan)
SP3-AQ2-01	VOCs, Inorganic gases (Carbon monoxide, Hydrogen, etc.)	MOS	FIS (Japan)
MP135A	Alcohols (Ethanol, etc.), Ketones (Acetone, etc.), Nitrogenous compounds (Ammonia, Cyanide, etc.), Benzene ring compounds (Benzene, Toluene, etc.)	MOS	Winsen (China)
MP4	Alcohols (Ethanol, etc.), Hydrocarbons (Methane, etc.), Sulfur compounds (Thioethers), Inorganic gases (Carbon monoxide, Hydrogen, etc.)	MOS	Winsen (China)
MP503	Alcohols (Ethanol, etc.), Aldehydes (Formaldehyde, Acetaldehyde, etc.), Hydrocarbons (methane, isobutane, etc.), Benzene ring compounds (Benzene, Toluene, etc.), Inorganic gases (Carbon monoxide, Hydrogen)	MOS	Winsen (China)
MP901	Alcohol, smoke, formaldehyde, toluene, benzene, acetone	MOS	Winsen (China)
MQ135	Nitrogenous compounds (Ammonia, Amines, etc.), Sulfur compounds (Thioethers, Hydrogen sulfide, etc.), Benzene ring compounds (Benzene, Toluene, etc.)	MOS	Winsen (China)
MQ136	Hydrocarbons (Methane, etc.), Alcohols (Ethanol, etc.), Inorganic gases (Carbon monoxide, Hydrogen, Hydrogen sulfide, etc.)	MOS	Winsen (China)
MQ137	Nitrogenous compounds (Ammonia, Amines, etc.), Alcohols (Ethanol, etc.)	MOS	Winsen (China)
MQ138	Alcohols, ketones, aldehydes, aromatic and other organic solvents	MOS	Winsen (China)
MQ3B	Alcohols (Ethanol, etc.)	MOS	Winsen (China)
WSP2110	Benzene ring compounds (Benzene, Toluene, etc.), Aldehydes (Formaldehyde, etc.), inorganic gases (Hydrogen, etc.)	MOS	Winsen (China)
MS1100	Toluene, benzene, formaldehyde, VOCs	MOS	Ogam (Korea)
GSBT-11	VOCs, toluene, benzene, formaldehyde	MOS	Ogam (Korea)
TGS4161	Carbon dioxide	Electrochemistry	Figaro (Japan)
NH3-3E100SE	Ammonia	Electrochemistry	CITY (UK)
4OXV	Oxygen	Electrochemistry	CITY (UK)
4S	Sulfur dioxide	Electrochemistry	CITY (UK)
4HS	Hydrogen sulfide	Electrochemistry	CITY (UK)
CH20/M-10	Formaldehyde	Electrochemistry	Membrapor (Switzerland)
4CH3SH-10	Methyl mercaptan	Electrochemistry	Solidsense (Germany)

**Table 2:** Number of samples in each dataset.

Number of Samples		Bacteria 1	Bacteria 2	Bacteria 3	Bacteria 4	Bacteria 5	Total
**Dataset 1**	train samples	67	46	57	66	46	282
	test samples	29	20	25	28	19	121
**Dataset 2**		87	71	83	84	76	401

**Table 3:** Procedure of SAIS algorithm.

SAIS algorithm:
**Input:**
Dataset **X**_S_
Dataset **X**_T_
**Output:**
The label of **X**_T_.
**Procedure:**
1. Transform every sample of source domain to a *d*-dimensional *z*-normalized vector;
2. Analyze the transformed source data by PCA and obtain the *D* eigenvectors corresponding to the *D* largest eigenvalues (i.e., **S**_S_);
3. Transform the source data to the source subspace, $X_{\mathrm{STr}}=X_{S}S_{S}$;
4. Transform every sample of target domain to a *d*-dimensional *z*-normalized vector;
5. Analyze the transformed target data by PCA and obtain the *D* eigenvectors corresponding to the *D* largest eigenvalues (i.e., **S**_T_);
6. Calculate the transformation matrix M^*^ by Equation (5) and further calculate the source-aligned target coordinate system: $S_{a}=S_{T}M^{*}=S_{T}{\left(S_{T}\right)}^{T}S_{S}$;
7. Transform the target data to the new coordinate system, $X_{\mathrm{TtoS}}=X_{T}S_{a}=X_{T}S_{T}{\left(S_{T}\right)}^{T}S_{S}$;
8. Use $X_{\mathrm{STr}}$ to train the prediction model C;
9. Predict the label of $X_{\mathrm{TtoS}}$ by the prediction model C.

**Table 4:** Recognition rate (%) for each method.

Method	Dataset 1	Dataset 2	Bacteria 1	Bacteria 2	Bacteria 3	Bacteria 4	Bacteria 5
ELM	100	29.18	62.07	0	37.35	71.43	1.32
SVM	99.01	26.68	28.74	1.41	0	96.43	0
PCA_ELM_	100	27.43	39.08	23.94	25.3	21.43	26.32
PCA_SVM_	94.04	22.94	60.92	12.68	14.46	4.76	18.42
OSC_ELM_	96.97	21.35	2.99	21.69	74.7	0	5.26
DRCA	95.46	29.68	51.72	0	38.55	41.67	9.21
DC-AELM(10)	98.26	29.18	33.34	0	39.76	65.48	0
DC-AELM(20)	100	31.18	48.28	0	38.56	57.15	3.95
DC-AELM(30)	100	33.17	42.53	15.50	39.76	54.77	7.90
DAELM-S(10)	99.35	54.16	91.03	50.7	29.88	41.67	55.53
DAELM-S(20)	100	70.02	85.28	63.94	51.8	77.85	69.47
DAELM-S(30)	100	79.21	93.1	73.7	70.68	**88.09**	67.98
DAELM-T(10)	98.51	55.21	81.61	66.19	37.1	42.38	48.68
DAELM-T(20)	100	69.16	77.78	80.28	52.61	81.35	53.5
DAELM-T(30)	100	79.8	93.1	78.4	73.89	83.3	68.42
SAIS(5)	93.30	46.64	75.87	40.85	22.90	45.24	46.06
SAIS(10)	94.54	56.61	86.21	63.38	34.94	55.96	40.79
SAIS(15)	100	62.60	89.66	59.16	36.15	60.72	65.79
SAIS(20)	100	75.07	90.81	71.84	56.63	**84.53**	69.74
SAIS(25)	100	79.56	**93.11**	**83.10**	69.88	79.77	71.06
SAIS(30)	100	**82.55**	91.96	80.28	**74.70**	**84.53**	**80.27**
